# A Mental Models Approach to Assessing Public Understanding of Zika Virus, Guatemala

**DOI:** 10.3201/eid2405.171570

**Published:** 2018-05

**Authors:** Brian G. Southwell, Sarah E. Ray, Natasha N. Vazquez, Tere Ligorria, Bridget J. Kelly

**Affiliations:** RTI International, Research Triangle Park, North Carolina, USA (B.G. Southwell, N.N. Vazquez);; RTI International, Atlanta, Georgia, USA (S.E. Ray);; RTI International, Guatemala City, Guatemala (T. Ligorria);; RTI International, Washington, DC, USA (B.J. Kelly)

**Keywords:** Zika virus, mental models, public understanding, risk communication, science communication, mosquitoborne diseases, viruses, Guatemala, vector-borne infections

## Abstract

Mental models are cognitive representations of phenomena that can constrain efforts to reduce infectious disease. In a study of Zika virus awareness in Guatemala, many participants referred to experiences with other mosquitoborne diseases during discussions of Zika virus. These results highlight the importance of past experiences for Zika virus understanding.

Current risk communication literature includes guidelines regarding crises (*1*,*2*). Creating and distributing risk-mitigation information amid a nexus of emotion, public health threats, a journalistic tendency toward sensationalism, and misinformation can be daunting (*3*,*4*), which can make general guidelines appealing. We argue here, though, that tension between widely held, preexisting mental models of disease and the circumstances of each new emergent infectious disease offers an underappreciated challenge. By better acknowledging how existing audience mindsets reflect past experiences sometimes at odds with new circumstances, we can move beyond set guidelines to call for formative research, psychologically oriented literature reviews, and social discourse monitoring as crucial steps to address emerging infectious diseases. To support our argument, we offer case evidence regarding public understanding of Zika virus in Guatemala in early 2016.

Any assessment of public health intervention potential can begin with understanding existing mental models among a population. Mental models involve how persons imagine and conceptualize phenomena (*5*,*6*; Technical Appendix). In the case of infectious disease, mental models can include conceptualization of disease transmission routes and processes and constraints, just as mental models of mechanical tools can include a person’s understanding of engineering principles.

To clarify how Guatemalans perceived Zika virus in spring 2016, we conducted 8 focus groups (with separate groups for men and women) and 10 in-depth individual interviews (coordinated in country by T.L.). Participants were adults 18–49 years of age in both urban and rural regions affected by the virus. We recruited participants from the database of a market research firm in Guatemala (ConsuMer). Staff conducted all focus groups and interviews in Spanish in 2 departments (Zacapa and Suchitepéquez) (*7*); focus groups took place in central locations (e.g., restaurants) and individual interviews occurred in participants’ homes.

The importance of personal experience with mosquitoborne viral disease in informing Zika virus understanding was striking. Virtually all participants were aware of Zika as a disease affecting Guatemala at the time of the interviews. At the same time, much of the discussion with participants clearly referred to other mosquitoborne diseases, rather than their conceiving of Zika virus as a new pathogen.

This pattern is understandable given the disease context in Guatemala, where dengue fever has been endemic for years, and given the substantial outbreak of chikungunya disease in 2014. Zika virus emerged and began to spread rapidly in Guatemala in late 2015. Participants apparently drew on experience with dengue and chikungunya as a baseline in understanding Zika virus. Mosquitoes were most commonly mentioned as a vector for the Zika virus, and participants often pointed out that the type of mosquito that transmitted Zika also was responsible for chikungunya and dengue.

Some participants made distinctions between mosquitoborne diseases. Many participants (>1 in each of all 8 focus groups and 6 in individual interviews), for example, noted that the presence of conjunctivitis (red eyes) distinguishes Zika from other mosquitoborne diseases. Even in such cases, however, dengue and chikungunya served as an anchoring reference against which Zika virus was compared. Anchoring bias is a human tendency to rely on an initial piece of information even when new information comes to light (*8*).

Such predominant understanding of mosquitoborne transmission appears to have overshadowed other possibilities for transmission routes in popular imagination. Fewer than half of the in-depth interview participants reported knowing that Zika virus could be sexually transmitted. That gap in understanding poses challenges to preventive efforts to change social interaction (as opposed to emphasizing prevention of interaction with mosquitoes). Moreover, many participants (>1 in each of 6 focus groups and half in individual interviews) expressed some sense of inevitability regarding mosquitoborne disease, likely because avoiding mosquito bites in Guatemala at certain times of the year can be difficult.

Anecdotal experience with symptoms loomed large in discussion. Participants (>1 in each of 4 focus groups) noted, after learning about sexual transmission possibilities, that they would look for symptoms in a partner and base sexual behavior on their assessment (insofar as they had agency to decide). We know, however, that a person infected with Zika virus might not have easily observable symptoms. Even if persons accept the possibility of sexual transmission, they might not engage in safe sex practices with asymptomatic infected partners.

If persons understand Zika virus through a mental model informed by dengue or chikungunya, public health officials should address potential confusion, especially in light of differences (e.g., sexual transmission) that might be misunderstood or ignored. Even when not confusing the illnesses, participants clearly conceptualized Zika in comparison with relatively more familiar illnesses. In this way, they operated in similar fashion as consumers encountering novel products do (*9*,*10*). Public health messaging might leverage this tendency. If it is easiest to understand a new outbreak in comparison to a previous one, using analogy or direct comparison might be effective but will also require careful emphasis on what is new.

Technical Appendix Mental models as a foundation for public communication.
