# Supplementary material for: A Mental Models Approach to Assessing Public Understanding of Zika Virus, Guatemala
Source: Emerg Infect Dis. 2018 May;24(5):938–9. doi: 10.3201/eid2405.171570 (PMC5938769; doi:10.3201/eid2405.171570)
Supplement: Technical Appendix — Mental models as a foundation for public communication. [file 17-1570-Techapp-s1.pdf]

Article DOI: https://doi.org/10.3201/eid2405.171570

A Mental Models Approach to Assessing Public Understanding of Zika Virus, Guatemala

Technical Appendix

Mental Models as a Foundation for Public Communication

Cognitive psychology and brain sciences researchers developed the concept of a mental model to explain how people make sense of, or mentally represent, processes and physical entities when considering them. Because we apply the mental model concept to public understanding of Zika virus in Guatemala in our letter, some readers might find a brief summary of thinking about mental models as a tool for risk communication to be helpful.

Mental models are cognitive representations that comprise how people imagine and conceptualize a phenomenon (*1–4*). For example, people might understand a house to be a structure with a roof and doors and windows in which people live; they also might have ideas about appropriate building materials or the ways in which a roof physically operates to protect and comfort occupants. The complexity and dimensions of a person's mental model of a phenomenon can constrain engagement with information about that phenomenon. Atman and colleagues (*5*) cite an example of how mental models can constrain popular understanding of the environment: they found evidence for some misunderstanding of the effects of radon gas in homes that stemmed from participants' models of radon. Some people mistakenly viewed radon as a long-lasting (as opposed to relatively short-lived) gas, which undermined their sense of the utility of efforts to clear radon from a house as a way of mitigating problems associated with radon. Viewing radon as a long-lasting or permanent contaminant could discourage people from taking action because of fatalism or perceived inability to combat the contamination.

In the case of infectious disease, mental models can include conceptualization of disease transmission as well as perceptions of the mechanisms through which the disease causes effects, as we note in our research letter. Importantly, conceptualizations of disease that people develop

based on their own observations, experiences, and background knowledge can differ substantially from models described in scientific, peer-reviewed literature (*6–10*): for example, some people view the common cold as being a function of cold weather rather than a viral infection (*9*) or some middle school students hold a mental model of vaccination as a process in which the vaccine attacks a virus that is already present within a body (*10*). Acknowledging audience members' foundational understanding of how processes like disease transmission work, in turn, can bolster prospects for risk communication efforts.

References

1. Craik K. The nature of explanation. New York: Cambridge University Press; 1967.

2. Johnson-Laird P. Mental models. Cambridge (MA): Harvard University Press; 1983.

3. Jones NA, Ross T, Lynam PP, Leitch A. Mental models: an interdisciplinary synthesis of theory and methods. Ecol Soc. 2011;16:art46. http://dx.doi.org/10.5751/ES-03802-160146

4. Morgan MG, Fischhoff B, Bostrom A, Atman C. Risk communication: a mental models approach. New York: Cambridge University Press; 2001.

5. Atman CJ, Bostrom A, Fischhoff B, Morgan MG. Designing risk communications: completing and correcting mental models of hazardous processes, part I. Risk Anal. 1994;14:779–88. PubMed http://dx.doi.org/10.1111/j.1539-6924.1994.tb00289.x

6. Chi MTH, Slotta JD, de Leeuw N. From things to processes: a theory of conceptual change for learning science concepts. Learn Instr. 1994;4:27–43. http://dx.doi.org/10.1016/0959-4752(94)90017-5

7. Duschl RA, Schweingruber HA, Shouse AW, editors. Taking science to school: learning and teaching science in grades K–8. Washington: National Academies Press; 2007.

8. Vosniadou S, Brewer W. Mental models of the earth: a study of conceptual change in childhood. Cognit Psychol. 1992;24:535–86. http://dx.doi.org/10.1016/0010-0285(92)90018-W

9. Sigelman CK. Age and ethnic differences in cold weather and contagion theories of colds and flu. Health Educ Behav. 2012;39:67–76. PubMed http://dx.doi.org/10.1177/1090198111407187

10. Jee BD, Uttal DH, Spiegel A, Diamond J. Expert-novice differences in mental models of viruses, vaccines, and the causes of infectious disease. Public Underst Sci. 2015;24:241–56. PubMed http://dx.doi.org/10.1177/0963662513496954
